# Hubs and Authorities in the World Trade Network Using a Weighted HITS Algorithm

**DOI:** 10.1371/journal.pone.0100338

**Published:** 2014-07-22

**Authors:** Tsuyoshi Deguchi, Katsuhide Takahashi, Hideki Takayasu, Misako Takayasu

**Affiliations:** 1 Department of Computational Intelligence and Systems Science, Interdisciplinary Graduate School of Science and Engineering, Tokyo Institute of Technology, Midori-ku, Yokohama, Japan; 2 Department of Economics, Kokugakuin University, Shibuya-ku, Tokyo, Japan; 3 Sony Computer Science Laboratories, Shinagawa-ku, Tokyo, Japan; 4 Graduate School for Advanced Mathematical Sciences, Meiji University, Tokyo, Japan; University Toulouse 1 Capitole, France

## Abstract

We investigate the economic hubs and authorities of the world trade network (WTN) from 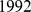 to 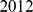, an era of rapid economic globalization. Using a well-defined weighted hyperlink-induced topic search (HITS) algorithm, we can calculate the values of the weighted HITS hub and authority for each country in a conjugate way. In the context of the WTN, authority values are large for countries with significant imports from large hub countries, and hub values are large for countries with significant exports to high-authority countries. The United States was the largest economic authority in the WTN from 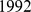 to 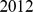. The authority value of the United States has declined since 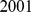, and China has now become the largest hub in the WTN. At the same time, China's authority value has grown as China is transforming itself from the “factory of the world” to the “market of the world.” European countries show a tendency to trade mostly within the European Union, which has decreased Europe's hub and authority values. Japan's authority value has increased slowly, while its hub value has declined. These changes are consistent with Japan's transition from being an export-driven economy in its high economic growth era in the latter half of the twentieth century to being a more mature, economically balanced nation.

## Introduction

Attempts to describe the patterns of international trade -who sells what to whom- have been a major preoccupation of international economists [1]. Studying the world trade network (WTN) is important not only for understanding the entire picture of world trade, but also for learning about the structure of the international economic system. With the globalization of the world economy in the last two decades, the structure of world trade has changed dramatically. World trade as a whole is increasing: the total value of world trade has quadrupled in the last two decades. However, the value of trade among advanced nations has grown only moderately, if at all. For example, trade between Japan and North American Free Trade Agreement (NAFTA) members has not grown at all during this period. However, trade between advanced countries and emerging countries has increased sharply. For instance, the value of trade between China and the European Union (EU) in 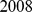 was five times larger than it was 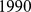 [2]. Newly emerging economies, including those of China, Korea, and members of the Association of South East Asian Nations (ASEAN), have become driving forces of expanding world exports. China has become the largest exporter in the WTN, replacing the United States. Is the United States losing its influence as China gains more power in the WTN? If so, on what evidence we can make such a determination?

In economic study, the word “hub” is not strictly defined, but it is conventionally used to indicate countries that have a large number of outgoing links with a large amount of trade - that is, they export large amounts of goods and services to other countries. An economic hub country typically imports raw materials, parts, and intermediate goods from other countries, assembles final goods, and exports these goods to consumer-driven countries. For example, Japan was a typical economic hub country in the 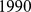
s, importing raw materials and exporting automobiles and electronics to the United States and Europe. Now, China seems to have become the world's foremost economic hub. We examine these assertions by applying the concepts of hub and authority used in social network analysis.

Social network studies have been conducted on the structure of international trade networks since World War II. The original idea of a world trade network can be traced to the studies of the League of Nations in 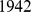, which employed graphs called *sociograms* [3], [4]. Recently, *sociograms* have been utilized numerically and computationally in the field of complex network analysis [5], [6]. In 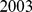, Serrano and Boguñá presented the first empirical characterization of the world trade web as a network built upon the trade relationships between countries throughout the world [7]. Since then, economists, social network scholars, and physicists have explored the structure and properties of the WTN [8]–[18]. In the WTN, most countries are characterized by weak trade links, but there exists a group of countries featuring a large number of strong relationships, which suggests a core-periphery structure [13]. This is particularly true of advanced countries. A major topic of research in recent years has been the investigation of the effects of ranking algorithms on the performance and behavior of networked systems. In 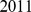, Ermann and Shepelyansky found that the PageRank approach gives a ranking that is independent of the trade amount of a given country [19]. Wei and Liu recently employed the weighted hyperlink-induced topic search (HITS) algorithm to characterize the world trade network [20]. HITS was originally introduced by Kleinberg to rate the importance of a node in a complex directed network using authority and hub values [21], [22]. In its original meaning, a node with a high authority value is pointed to by many other nodes with high hub values, and a node with a high hub value points to many nodes with high authority values. This algorithm has been used as a tool for the characterization of the network structure of homepages, and it has been applied to various economic areas, such as the analysis of money-flow networks among Japanese business firms [23], [24]. Using the 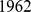
–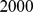 trade dataset compiled by the United States' National Bureau of Economic Research (NBER), Wei and Liu concluded that the “impact ranking” (equivalent to authority ranking) of the United States was decreasing and that the influence of China and Japan was increasing up to 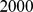 [20].

In this paper, we introduce the quantitative measures of the hub to clarify the WTN's topology changes more exactly in terms of network science. To do this, we purchase and analyze the International Monetary Fund's (IMF) Direction of Trade Statistics dataset for the period 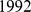
–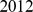 [25]. In the WTN, countries are nodes and the links are the exports and imports among them, where the weight of a link is defined as the annual amount of trade. Following the development of complex network studies, we introduce two definitions of “hub”: the “HITS hub,” which characterizes the network without weights, and the “weighted HITS hub,” which considers the weights. With these mathematically defined hubs, we can quantitatively discuss the WTN's economics hubs and their changes over time. As an export and an import always make a pair, we also introduce “HITS authority” and “weighted HITS authority” as counterparts of the concepts of the “HITS hub” and “weighted HITS hub” to characterize the role of imports in the WTN. Note that these network quantities generally take large values for large countries; however, they are not simply proportional to the amount of exports or imports: for some countries, these quantities take larger values depending on the WTN location.

## Measuring Influence in WTN

### Dataset

The data used in our study were compiled from the Direction of Trade Statistics (DOTS) by the International Monetary Fund. All the data can be downloaded or purchased in the form of CD-ROM from the official web site of the IMF [25]. DOTS provide information on bilateral trade relationship, exports and imports, between any pairs of countries or regions monthly and yearly from 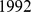 to 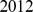 measured in US dollar. Theoretically the amount of export of country A to country B should be the same as the amount of import of country B from country A, however, there are small differences in these numbers. In the following data analysis we show results calculating all quantities based on the export yearly data as we checked that the results do not change by calculating based on the import yearly data.

The DOTS data includes regions like Europe and Africa, however, we pay attention only to countries. Some countries were newly founded and some had disappeared because of political upheaval during this period. Technically speaking, we treated these disappeared or newly established countries in the following way. In the process of compiling our dataset, we eliminated the entries of countries which had already gone before 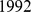 even though they still survive nominally in the original data. They are East Germany, USSR, Yemen Arab Republic and PD Yemen. We treated Belgium and Luxemburg separately since 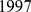 because they were consolidated before that in the IMF data. In the same way we dealt with Czech Republic and Slovakia Republic separately since 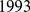. Former Yugoslavian countries were treated as well in the same way. Through these procedures we identified 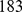
–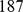 countries in each year which indicates the number of nodes in our paper.

### PageRank Algorithm and HITS Algorithm

In this section, we show the algorithms used in this study. First, to describe the method, we define the weighted network matrix, 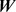, whose component, 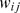, represents the annual amount of exports of goods (products) from country 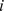 to country 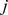 measured in millions of US dollars. By definition, 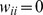. We also introduce the binary network matrix, 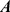, whose component 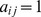 when 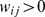 and 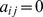 when 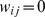.

We introduce PageRank, which characterizes the importance of a node in the network by considering random diffusion [26], [27]. PageRank is defined as the eigenvector of the following Google matrix:
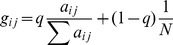
(1)


Here, 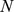 is the number of nodes and 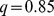 is a positive parameter introduced to ensure the existence of a unique solution by the Perron-Frobenius theorem. Denoting the Google matrix by 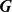, the PageRank vector, 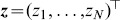, is obtained by repeating the following iteration until convergence:
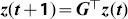
(2)


where, 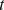 is the number of iterations, the super-script 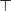 denotes the transpose of a vector or a matrix, and the initial condition is given by 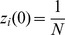 for all 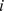. We also introduce a modified PageRank, the weighted PageRank, which is defined in the same way by replacing 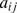 with 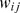 in equations (1) and (2).

Next, we introduce the HITS algorithm to define the HITS hub and authority. The HITS authority vector, 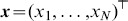, and the HITS hub vector, 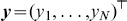, are defined by the limit of the following set of iterations:
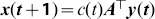
(3)

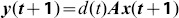
(4)


where 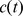 and 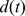 are normalization factors to make the sums of all elements become unity,
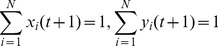
(5)


The initial values of the iterations are 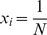 and 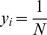 for all 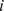. As is known from this formulation, the HITS authority vector, 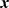, is the eigenvector for the matrix 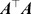; similarly, the HITS hub vector, 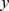, is the eigenvector for the matrix 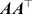. As the matrix elements of 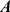 are non-negative, the elements of 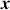 and 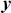 are also non-negative. As is known from this definition, a node has a larger value of HITS authority if it has more links from those nodes with larger HITS hub values; similarly, a node has a larger value of HITS hub if it is linked to those nodes with larger HITS authority values. The weighted HITS hub and weighted HITS authority are defined in exactly the same way, replacing 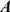 with 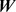 in Eq. (3) and (4).

We also define the shares of export/import for comparison with these hub/authority values by the following ratios: the amount of export/import divided by the whole amount,
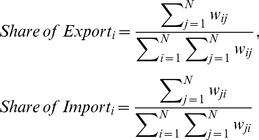
(6)


### Feasibility of the weighted HITS in the WTN

**Figure 1:**
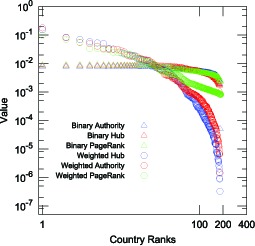
PageRank and Hub/Authority. Comparison of distributions of various network quantities in the year 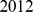. Authority (blue), Hub (red), PageRank (green); the binary cases and the weighted cases are represented by triangles and circles, respectively.

First, we show that the weighted HITS algorithm is more appropriate than the original HITS algorithm. Focusing on the top 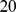 exporter countries in the WTN, we easily find that nearly all of these countries have connected with each other in the 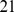-year period. This means that the top 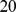 nodes comprise an almost-complete network. As a result, the HITS hub and HITS authority values calculated by the original HITS algorithm vary little among these top countries. For example, Korea and the United States are characterized by similar HITS values, although the trade export magnitude of the United States is more than 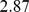 times greater than that of Korea. In Figure 1, we show the rank plots of the values calculated by the original HITS algorithm and the weighted HITS algorithm of the WTN for the year 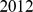. We find that the original HITS algorithm fails to represent the differences among the top 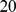 countries, while the weighted HITS algorithm reflects their differences. We judge that the weighted HITS values are a better characterization than the original HITS values.

The same discussion holds for the original PageRank algorithm and the weighted PageRank algorithm. In the case of WTN, neglect of the weights makes the top-ranked countries nearly identical, which is not favorable for WTN characterization. Comparing the weighted HITS and weighted PageRank in the case of WTN, the weighted HITS is more nontrivial, as it reflects the two-link network structure, while the weighted PageRank only reflects the one-link network structure. Therefore, in this paper, we focus on the weighted HITS as a mathematical definition of economic hub and authority.

Here, we discuss the query dependency of the HITS algorithm, as some papers indicate that PageRank is better than HITS because HITS is a query-dependent algorithm and PageRank is a query-independent algorithm [19], [28]. Query dependency is an important concept in homepage ranking algorithms. It means that the scheme assigning a score to a node depends on the context of the homepage network. In the case of WTN, we analyze all the nodes and links, as the number of nodes is only about 200. Thus, we analyze the network with the HITS algorithm globally, and there is no query-dependent problem in this case.

### WTN data for the past 21 years

**Figure 2:**
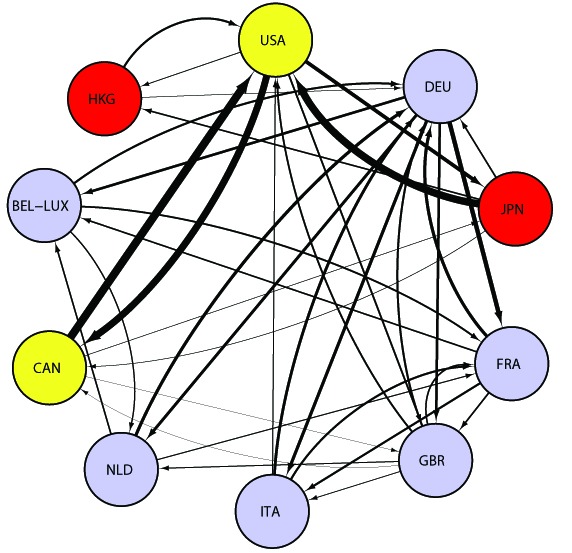
WTN among the top 10 countries in 1992. For each country, the top three exports/imports are indicated by the arrows. The countries are placed clockwise in order of total annual export, beginning with the United States. Colors specify the regions; the North America (yellow), Europe (blue), and East Asia (red). Abbreviations are as follows, “USA  =  United States”, “DEU  =  Germany”, “JPN  =  Japan”, “FRA  =  France”, “GBR  =  United Kingdom”, “ITA  =  Italy”, “NLD  =  Netherlands”, “CAN  =  Canada”, “BEL-LUX  =  Belgium-Luxembourg” and “HKG  =  Hong Kong.”

**Figure 3:**
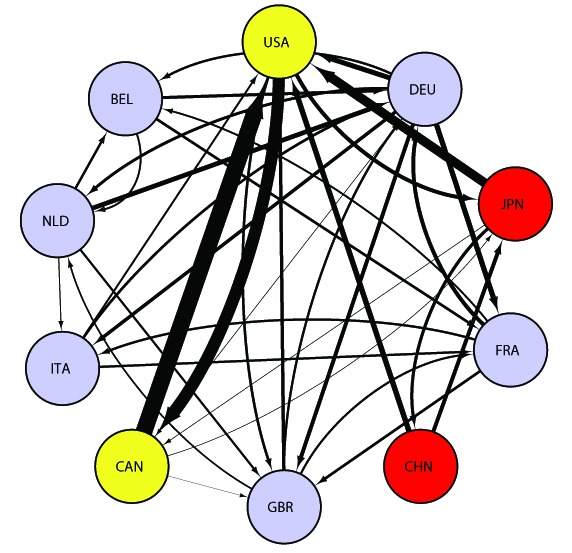
WTN among the top 10 countries in 2002. This figure follows the same format as Figure 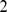. Abbreviations are as follows, “USA  =  United States”, “DEU  =  Germany”, “JPN  =  Japan”, “FRA  =  France”, “CHN  =  China”, “GBR  =  United Kingdom”, “CAN  =  Canada”, “ITA  =  Italy”, “NLD  =  Netherlands” and “BEL  =  Belgium.”

**Figure 4:**
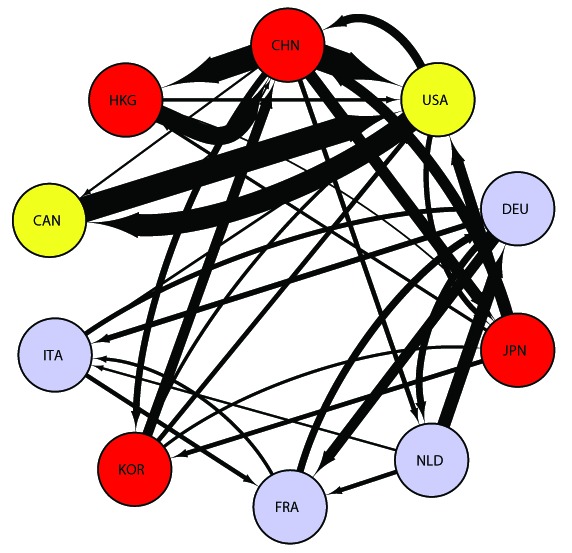
WTN among the top 10 countries in 2012. This figure follows the same format as Figure 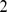. Abbreviations are as follows, “CHN  =  China”, “USA  =  United States”, “DEU  =  Germany”, “JPN  =  Japan”, “NLD  =  Netherlands”, “FRA  =  France”, “KOR  =  Republic of Korea”, “ITA  =  Italy”, “CAN  =  Canada” and “HKG  =  Hong Kong.”

By examining the WTN over time, we can observe its evolution. Figure 2 shows the trade relationships among the top 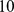 exporters in the world in 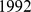. The width of the links is weighted by the export value in US dollars. The wider the links, the larger the exports. Apparently, there exist strong trade relationships among European countries (Great Britain, France, Germany, Belgium-Luxembourg, the Netherlands, and Italy). Strong ties between the United States and Japan and between the United States and Canada can also be observed. It is evident that the nexus of world trade was located in the advanced Western nations in 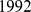. Until the Soviet Union collapsed in December 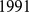, the world economic system was divided into East and West. Both Cold War powers were separated politically and economically for more than forty years. In 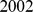, China appeared as a major exporter (see Figure 3). The rapid and historic economic growth of China began with its economic reform in December 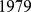. China's subsequent accession to the World Trade Organization (WTO) in 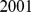 had a large impact on the world trade structure, and China's emergence has changed the global balance of economic and political power. The direction and composition of world trade are dynamic, reflecting the changing economic and political relations in international society. In 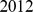, the differences between China and other countries became more distinct than they were in 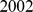 (see Figure 4). The competitiveness of products, industries, and firms affects a country's exports substantially. In 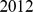, Korea became one of the world's top 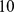 exporters because of its strengthened international competitiveness. With the emergence of China, Korea, and the ASEAN countries, it is obvious that the center of world trade has shifted toward Asia. In the debate on the rise and fall of nations, economists and policymakers have often highlighted global trade imbalances, especially the United States' huge trade deficit and the China's enormous trade surplus. Some have argued that the huge US trade deficit reflects the waning competitiveness of US products, and that the United States is losing its high status and influential position (as a *hegemon*, in the jargon of international politics) in the WTN.

### Hubs in the WTN

**Figure 5:**
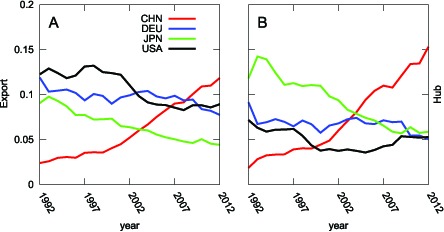
Transition of Exports Shares in the period 1992–2012 shown in panel (A). (B) Transition of the weighted HITS Hub Values in the period 1992–2012 for the four major countries: China (red), Germany (blue), Japan (green) and the United States (black). Accession to the WTO in 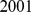 explains the sharp rise in China's weighted HITS hub value.

Here, we show the results and discuss the relationship between the weighted HITS hub values and the shares of the world exports for each country as listed in Table 1. We also discuss the changes in economic hub values shown in Table 2 and Figure 5, in which China was first in 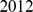 after being thirteenth in 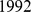. China's export value began to increase dramatically with its accession to the WTO in 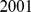. In particular, China's exports to the United States, the largest weighted HITS authority, have increased remarkably since 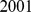. This explains the sharp rise in China's weighted HITS hub value. Korea rose from twelfth to seventh by strengthening its international competitiveness and supplanting Japanese exports throughout the global market. Japan's weighted HITS hub value was highest in 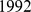, but it subsequently declined for the following reasons. 1) Exports from Japan to the United States and European countries were constrained by trade frictions. 2) Japanese firms emphasized production for local consumption instead of for exports. 3) Exports from Japan decreased because of the long-standing appreciation of Japan's currency [29]–[31]. Consequently, Japan's exports to countries with high weighted HITS authority values have become relatively small in the WTN. In contrast to China's rise, Japan's apparent fall reflects the so-called Lost Decade, during which Japan experienced a long-term economic downturn. Germany has shown a slight downward trend. The United States stagnated in the first half of the 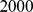
s, but the data suggest a possible recovery. The decline of the weighted HITS hub values of European countries is notable. The weighted HITS hub values of most European countries have decreased over the last two decades (see Table 2). This trend can be explained by the fact that exports from European countries have tended to concentrate within the European Union following the establishment of a common market. This is a well-known trade diversion effect.

**Table 1:** The Weighted HITS Hub Values and Shares of World Exports in 2012.

Country	Hub Value	Share in the World Exports
P.R. China	0.1532	0.1184
Canada	0.0842	0.0267
Mexico	0.0699	0.0217
Japan	0.0587	0.0442
United States	0.0529	0.0893
Germany	0.0504	0.0775
Republic of Korea	0.0343	0.0311
Hong Kong	0.0323	0.0255
Netherlands	0.0247	0.0373
Saudi Arabia	0.0239	0.0207
France	0.0227	0.0324
United Kingdom	0.0210	0.0239
Singapore	0.0198	0.0230
Italy	0.0197	0.0284
Belgium	0.0189	0.0250
India	0.0154	0.0171
Russian Federation	0.0142	0.0252
Australia	0.0141	0.0146
Switzerland	0.0133	0.0132
Brazil	0.0130	0.0138

**Table 2:** Weighted HITS Hub Value Ranks in 1992 and 2012 (top 20).

1992				2012		
Rank	Country	Value	Up or Down	Rank	Country	Value
1	Japan	0.1181	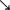	1	P.R. China	0.1532
2	Canada	0.0983	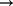	2	Canada	0.0842
3	Germany	0.0915	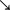	3	Mexico	0.0699
4	United States	0.0717	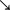	4	Japan	0.0587
5	France	0.0552	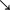	5	United States	0.0529
6	United Kingdom	0.0502	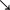	6	Germany	0.0504
7	Italy	0.0451	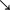	7	Republic of Korea	0.0343
8	Hong Kong	0.0376	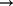	8	Hong Kong	0.0323
9	Netherlands	0.0375	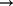	9	Netherlands	0.0247
10	Mexico	0.0350	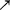	10	Saudi Arabia	0.0239
11	Belgium-Luxembourg	0.0335	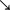	11	France	0.0227
12	Republic of Korea	0.0239	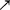	12	United Kingdom	0.0210
13	P.R. China	0.0185	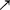	13	Singapore	0.0198
14	Switzerlad	0.0183	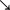	14	Italy	0.0197
15	Singapore	0.0178	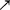	15	Belgium	0.0189
16	Spain	0.0160	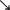	16	India	0.0154
17	Saudi Arabia	0.0149	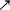	17	Russian Federation	0.0142
18	Sweden	0.0132	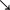	18	Australia	0.0141
19	Austria	0.0114	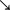	19	Swizerland	0.0133
20	Malaysia	0.0114	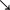	20	Brazil	0.0130

**Figure 6:**
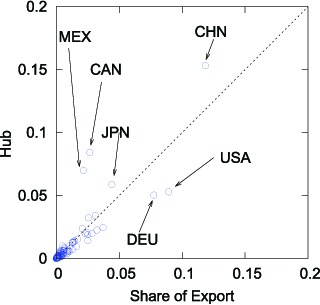
Comparison of the weighted HITS Hub Value and Trade Share in Global Exports in 2012.

Figure 6 demonstrates this relationship by showing the correspondence between the weighted HITS hub values and the trade share of each country in world exports. The horizontal axis indicates the shares of exports, while the corresponding hub values are plotted on the vertical axis. The scatter of points does not always cluster around the dotted 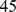-degree line (it is supposed to be on the 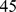-degree line if the hub values and the share are proportionate). This means that the weighted HITS hub values and the shares are proportional as the link structure of WTN affects the weighted HITS values in some way. While China's share of world exports is 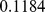 (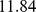
%), its hub value is 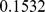, which is significantly larger than if it were in due proportion, which suggests that China is more influential in actuality than the trade statistics suggest.

### Authority in the WTN

**Figure 7:**
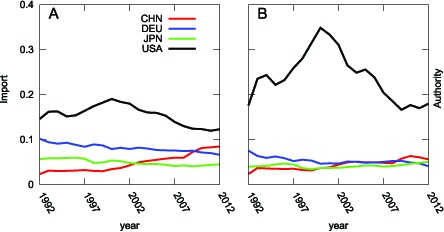
Transition of Imports Shares from 1992–2012 shown in panel (A). (B) Transition of Authority Values from 1992–2012 for the four major countries: China (red), Germany (blue), Japan (green) and the United States (black). The authority value of the United States increased in the latter half of the 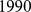
s because of the advent of the New Economy, which was driven by the IT revolution. It began to decline in the wake of the burst of the IT Bubble in 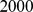.

Next, we examine the weighted HITS authority values and shares of imports in the WTN (see Tables 3 and 4 and Figure 7). The United States has maintained the highest value since 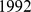. Hong Kong and China rank second and third, respectively, in 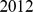. The weighted HITS authority value of the United States rose remarkably in the late 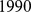
s during the economic boom known as the *New Economy*, which was led by the IT industry. Its weighted HITS authority value rose to a record high in 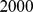, by far the largest in the WTN. It seemed that the United States had established *hegemon* status in the world political economy. However, its *hegemony* has weakened since 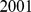, while emerging countries have experienced dramatic economic growth. Although the United States has maintained its status as the most influential country in the WTN up to the present, its dominance is not as overwhelming as before (see Figure 7).

**Table 3:** The Weighted HITS Authority Values and Shares of World Imports in 2012.

Country	Authority Value	Share in the World Imports
United States	0.1797	0.1229
Hong Kong	0.0816	0.0355
P.R. China	0.0563	0.0847
Japan	0.0507	0.0451
Germany	0.0408	0.0663
Republic of Korea	0.0336	0.0288
Netherlands	0.0323	0.0381
United Kingdom	0.0321	0.0385
Canada	0.0318	0.0260
France	0.0280	0.0397
Mexico	0.0257	0.0195
India	0.0197	0.0246
Italy	0.0184	0.0274
Singapore	0.0183	0.0187
Belgium	0.0176	0.0240
Russian Federation	0.0174	0.0177
Australia	0.0155	0.0131
Brazil	0.0149	0.0133
Thailand	0.0141	0.0125
Malaysia	0.0139	0.0122

**Table 4:** Weighted HITS Authority Value Ranks in 1992 and 2012 (top 20).

1992				2012		
Rank	Country	Value	Up or Down	Rank	Country	Value
1	United States	0.1759	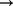	1	United States	0.1797
2	Germany	0.0754	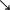	2	Hong Kong	0.0816
3	France	0.0637	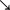	3	P.R. China	0.0563
4	United Kingdom	0.0551	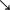	4	Japan	0.0507
5	Canada	0.0460	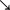	5	Germany	0.0408
6	Netherlands	0.0453	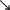	6	Republic of Korea	0.0336
7	Italy	0.0449	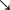	7	Netherlands	0.0323
8	Belgium-Luxembourg	0.0411	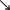	8	United Kingdom	0.0321
9	Japan	0.0398	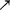	9	Canada	0.0318
10	Hong Kong	0.0274	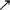	10	France	0.0280
11	Spain	0.0266	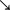	11	Mexico	0.0257
12	P.R. China	0.0236	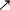	12	India	0.0197
13	Switzerland	0.0233	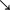	13	Italy	0.0184
14	Republic of Korea	0.0229	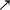	14	Singapore	0.0183
15	Mexico	0.0222	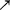	15	Belgium	0.0176
16	Austria	0.0186	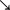	16	Russian Federation	0.0174
17	Singapore	0.0180	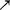	17	Australia	0.0155
18	Australia	0.0124	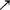	18	Brazil	0.0149
19	Thailand	0.0116	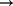	19	Thailand	0.0141
20	Sweden	0.0115	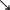	20	Malaysia	0.0139

**Figure 8:**
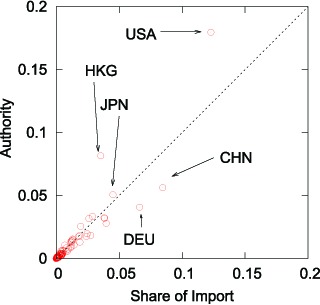
Comparison of the weighted HITS Authority Value and Trade Share in Global Imports in 2012.

Now we examine the relationship between a country's weighted HITS authority value and its share of world imports. Figure 8 shows that the data points do not fall on the 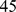-degree line. We find that the weighted HITS authority and the share are not necessarily proportional, just as in the hub case. While the United States' share of world imports is 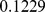 (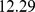
%), its weighted HITS authority value is 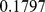, which is significantly larger than if it were in due proportion, implying that the United States is more influential than the trade statistics would suggest (see Table 3).

### Suggested explanation for the Hub-Authority correlation

**Figure 9:**
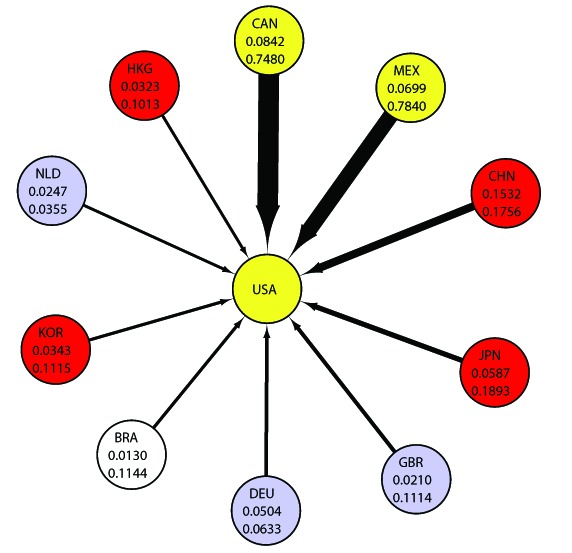
Major Exporters to the United States; Hub Values and Export Shares. The upper number is the weighted HITS hub value and the lower number is the share of exports to the United States. Abbreviations are as follows, “CAN  =  Canada”, “MEX  =  Mexico”, “CHN  =  China”, “JPN  =  Japan”, “GBR  =  United Kingdom”, “DEU  =  Germany”, “BRA  =  Brazil”, “KOR  =  Republic of Korea”, “NLD  =  Netherlands” and “HKG  =  Hong Kong” and “USA  =  United States.”

**Figure 10:**
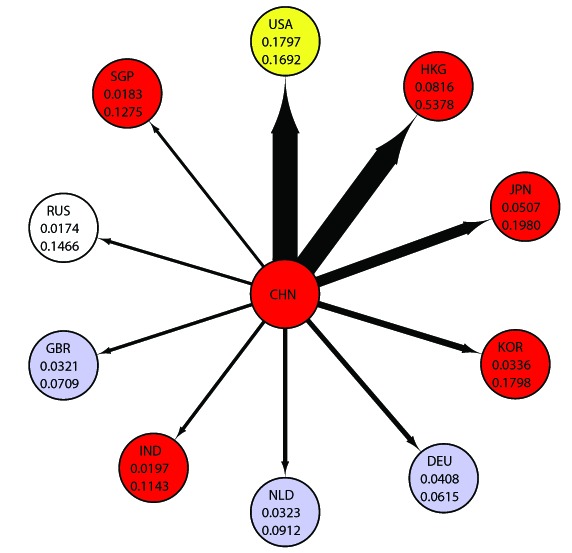
Major Export destinations of China; Authority Values and Import Shares. The upper number is the weighted HITS authority value and the lower number is the share of imports from China. Abbreviations are as follows, “USA  =  United States”, “HKG  =  Hong Kong”, “JPN  =  Japan”, “KOR  =  Republic of Korea”, “DEU  =  Germany”, “NLD  =  Netherlands”, “IND  =  India”, “GBR  =  United Kingdom”, “RUS  =  Russian Federation” and “SGP  =  Singapore” and “CHN  =  China.”

A node with larger value of HITS authority is considered to be a large importing country in which demand for foreign goods (products) is significantly large, a big “consumer” in the world market. On the other hand, a node with larger value of HITS hub is interpreted to be a large exporting country which has a large capacity of supplying goods, a big “factory” in the world market. By definition value of HITS authority and HITS hub are interrelated. They are strengthening and reinforcing each other. The higher the hub, the higher the authority, and vice versa. Consequently, the hub value and authority value become higher than our simple observation on exporting share of each country to a trading partner (see Figure 9 and 10).

**Figure 11:**
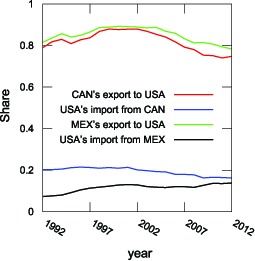
Changes of Export and Import Shares from Canada/Mexico to the United States. Export Share means the share of Canada/Mexico's exports to the United States and Import Share means the share of the United States' imports from Canada/Mexico.

Firstly, we examine the relation to the highest weighted authority country, the United States. In this case, Canada and Mexico offer a good example as exporters. Their shares of the global exports are just 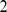
–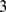
% percent, but their economic hub values are disproportionately high. In terms of weighted HITS hub score, Canada remained the second highest in the ranking, while Mexico rose from tenth to third with the establishment of NAFTA in 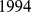, which gave Canada and Mexico easier access to the market of the United States, the country with the high weighted HITS authority value in the world. This means that Canada and Mexico are in a special position because of their relations with the United States. This reflects the fact that the weighted HITS hub values are affected by the network structure of world trade. Figure 9 lists the major exporting countries to the United States along with their weighted HITS hub values (upper number) and percentage of exports to the United States (lower number). For instance, Canada's weighted HITS hub score is 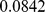, and 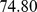
% of its exports are to the United States. Mexico's weighted HITS hub score is 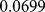, and 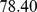
% of its exports are to the United States. With this figure, we can verify that Canada's and Mexico's shares of exports to that of the United States are remarkably higher than other countries'. We also find that this tendency has held for 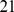 years, as demonstrated in Figure 11.

**Figure 12:**
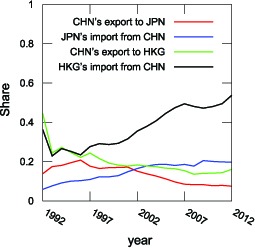
Changes of Export and Import Shares from China to Hong Kong/Japan. Export Share means the share of China's exports to Hong Kong/Japan and Import Share means the share of Hong Kong/Japan's imports from China.

On the other hand, we check the relation to the highest weighted HITS hub country, mainland China. In this case, Hong Kong offers a good example as an importer. We can elucidate this fact more clearly by comparing Hong Kong to Japan. Hong Kong's weighted HITS authority value is 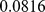, while its import share is only 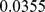. Japan's weighted HITS authority value is 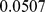 and its import share is 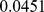. What causes the differences? The share of imports from China is the answer (Figures 10 and 12). Figure 10 shows the weighted HITS authority of China's major export countries and the percentage of imports from China and Figure 12 shows the time evolution of Hong Kong's and Japan's import shares for overall trade from China. From these figures, we can confirm that Hong Kong's import share from China changed drastically in 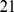 years. During this period, Hong Kong's authority ranking position increased from tenth to second (see Table 4). On the other hand, in the case of Japan, the moderate change of its share raised its ranking from ninth to fourth (see Table 4). Hong Kong's rapid change implies that it has taken advantage of transit trade. In other words, Hong Kong has grown into a China's most important export channel to the world.

From these examples, we find that the weighted HITS hub and authority values are larger than the shares of the export/import ratio in each country if the main contributor's conjugate weighted HITS value is high.

### Emergence of China

As our final topic, we consider the changes of China. The most striking change over the last two decades in the WTN has been the rise of China. China has become the largest weighted HITS hub country. Hong Kong and China ranked second and third respectively (and Macao is eighty-second), for authority in 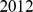. This means that many countries with high hub values have increased their exports to Hong Kong and China as both have become able to afford to import huge amounts of foreign goods. China has increased its purchasing power for foreign goods because of the rapid increase of its national income. In other words, China has become one of the world's most important markets.

**Figure 13:**
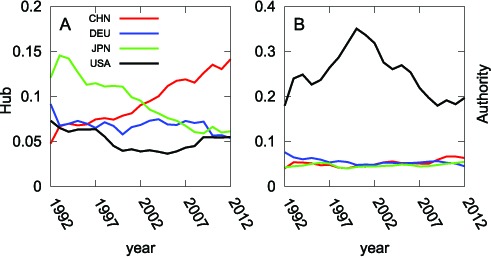
Hub and Authority Values of Greater China after Merging Hong Kong and Macao into China.

What if we put China, Hong Kong and Macao together in order to observe the hub and authority of greater China? Figure 13 shows the transition of the weighted HITS hub (13A) and authority (13B) after merging China, Hong Kong and Macao. Comparing Figures 13A and 5B, the combined hub value is nearly twice as high as the export value in the 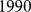
s and almost same in the 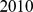
s. Furthermore, the weighted HITS authority value does not change much after consolidating their trade data (see Figures 13B and 7B). This is because bilateral trade between China and Hong Kong is very large and their internal trade has a greater effect on them than on other countries. Thus the large amount of trade between China and Hong Kong cancels out when the two nodes are merged.

## Conclusion

We introduced the weighted HITS hub and authority as mathematical definitions of the economic hub and authority. With these values, we elucidated the changes of WTN over the last 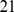 years. The United States has been the largest economic authority in the global trade network and the largest destination of world exports. The authority value of the United States increased in the latter half of the 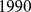
s because of the advent of the New Economy, which was driven by the IT revolution. It began to decline in the wake of the burst of the IT Bubble in 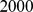. On the other hand, China has changed the structure of the WTN, particularly since 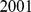, by dramatically increasing its trade volume and number of trading partners. China's rise is reflected in the weighted HITS network analysis. China now has the world's largest hub value and the third largest authority value. In terms of changes of its hub and authority value, China is transforming itself from the “factory of the world” to the “market of the world.” Korea's authority value places it in the top 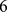, reflecting its increased national income and high economic growth. European countries fell in the ranking of authority values, reflecting the increased difficulty for outsiders to export to EU countries: EU countries benefit from free trade by non-tariff or preferential tax schemes within the EU region, but outsiders cannot. Thus, EU countries have tended to trade more with other EU countries. This is called the trade diversion effect, a consequence of regional economic integration. Japan's authority value increased slowly in 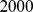
–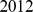, but its hub value declined. These changes are consistent with Japan's change from an export-driven economy in its high economic growth era in the latter half of the twentieth century to a more mature, more balanced nation. Mexico and Canada have larger hub values than linearly expected from their exports because of their relations with the United States. The hub value of Mexico grew rapidly in this era, indicating that NAFTA, which was established in 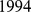, has had an impact on the amount of trade and the link structure. We also find that the Hong Kong's weighted authority becomes larger because of its relation with China. This suggests that Hong Kong has grown as into one of the most important export channels in the world. Hypothetically, the virtual integration of China, Hong Kong and Macao does not make a substantial impact on weighted values, and particularly has less impact on authority value. This is because the huge amount of trade between China and Hong Kong cancels out when the two nodes are merged.

In this paper we showed time evolution of world trade in terms of hub-authority, and we found that some of typical behaviors are consistently explained by changes in countries and international relations. As these changes are rather slow taking several years typically, it will be a promising next work to make a numerical model which can predict hub-authority values in the near future.

## References

[1] Krugman PR, Obstfeld M, Melitz M (2011) International Economics (9th Edition). Prentice Hall.

[2] Ministry of Economy, Trade and Industry of Japan (2011) White Paper on International Economy and Trade 2011.

[3] Benedictis LD, Tajoli L (2011) The World Trade Network. The World Economy 34: 1417–1454.

[4] League of Nations (1942) The World Trade Network. Princeton University Press.

[5] Newman M (2010) Networks: An Introduction. New York, NY, USA: Oxford University Press, Inc.

[6] Caldarelli G (2007) Scale-Free Networks: Complex Webs in Nature and Technology. Oxford University Press.

[7] Serrano MA, Boguñá M (2003) Topology of the world trade web. Phys Rev E 68: 015101. 10.1103/PhysRevE.68.015101 12935184

[8] Li X, Jin YY, Chen G (2003) Complexity and synchronization of the World trade Web. Physica A: Statistical Mechanics and its Applications 328: 287–296.

[9] Garlaschelli D, Loffredo MI (2004) Fitness-Dependent Topological Properties of the World Trade Web. Phys Rev Lett 93: 188701. 15525215 10.1103/PhysRevLett.93.188701

[10] Garlaschelli D, Loffredo MI (2005) Structure and evolution of the world trade network. Physica A: Statistical Mechanics and its Applications 355: 138–144.

[11] Garlaschelli D, Di Matteo T, Aste T, Caldarelli G, Loffredo MI (2007) Interplay between topology and dynamics in the World Trade Web. The European Physical Journal B 57: 159–164.

[12] Fagiolo G, Reyes J, Schiavo S (2009) World-trade web: Topological properties, dynamics, and evolution. Phys Rev E 79: 036115. 10.1103/PhysRevE.79.036115 19392026

[13] Fagiolo G, Reyes J, Schiavo S (2010) The evolution of the world trade web: a weighted-network analysis. Journal of Evolutionary Economics 20: 479–514.

[14] Barigozzi M, Fagiolo G, Garlaschelli D (2010) Multinetwork of international trade: A commodityspecific analysis. Phys Rev E 81: 046104. 10.1103/PhysRevE.81.046104 20481783

[15] He J, Deem MW (2010) Structure and Response in the World Trade Network. Phys Rev Lett 105: 198701. 21231202 10.1103/PhysRevLett.105.198701

[16] Barigozzi M, Fagiolo G, Mangioni G (2011) Identifying the community structure of the international-trade multi-network. Physica A: Statistical Mechanics and its Applications 390: 2051–2066.

[17] Squartini T, Fagiolo G, Garlaschelli D (2011) Randomizing world trade. I. A binary network analysis. Phys Rev E 84: 046117. 10.1103/PhysRevE.84.046117 22181237

[18] Squartini T, Fagiolo G, Garlaschelli D (2011) Randomizing world trade. II. A weighted network analysis. Phys Rev E 84: 046118. 10.1103/PhysRevE.84.046118 22181238

[19] Ermann L, Shepelyansky DL (2011) Google matrix of the world trade network. arXiv preprint arXiv:11035027.

[20] Wei W, Liu G (2012) Bringing Order to the World Trade Network. In: IPEDR Proceedings. Singapore: IACSIT Press, 28, p. 88.

[21] Kleinberg JM (1999) Authoritative sources in a hyperlinked environment. J ACM 46: 604–632.

[22] Easley D, Kleinberg JM (2010) Networks, Crowds, and Markets: Reasoning About a Highly Connected World. New York, NY, USA: Cambridge Univ Press.

[23] Ohnishi T, Takayasu H, Takayasu M (2009) Hubs and Authorities on Japanese Inter-Firm Network: Characterization of Nodes in Very Large Directed Networks. Progress of Theoretical Physics Supplement 179: 157–166.

[24] Miura W, Takayasu H, Takayasu M (2012) The origin of asymmetric behavior of money flow in the business firm network. The European Physical Journal Special Topics 212: 65–75.

[25] International Monetary Fund, Direction of Trade Statistics website. Available: http://www.imf.org/external/data.htm. Accessed: 2014 Jun 27.

[26] Brin S, Page L (1998) The anatomy of a large-scale hypertextual Web search engine. Computer Networks and ISDN Systems 30: 107–117.

[27] Page L, Brin S, Motwani R, Winograd T (1999) The PageRank Citation Ranking: Bringing Order to the Web. Technical Report 1999–66, Stanford InfoLab. Available: http://ilpubs.stanford.edu: 8090/422/.

[28] Henzinger M (2001) Hyperlink analysis for the Web. Internet Computing, IEEE 5: 45–50.

[29] Noland M (1995) US-Japan Trade Friction and its Dilemmas for US policy. World Economy 18: 237–267.

[30] Kawai M, Urata S (1998) Are trade and direct investment substitutes or complements? An empirical analysis of Japanese manufacturing industries. Economic development and cooperation in the Pacific Basin: Trade, investment, and environmental issues: 251–293.

[31] Takahashi K, Urata S (2010) On the use of FTAs by Japanese firms: further evidence. Business and Politics 12..

